# “Shot on Target”: A Case of an Unusual Peripheral Ossifying Fibroma of the Edentulous Upper Jaw Irritated by the Only Teeth of the Lower Jaw

**DOI:** 10.7759/cureus.47955

**Published:** 2023-10-30

**Authors:** Ioannis Fotopoulos, Vasileios Zisis, Eleftherios Anagnostou, Dimitrios Andreadis, Athanasios Poulopoulos, Theodoros Lillis, Nikolaos Dabarakis

**Affiliations:** 1 Dentoalveolar Surgery, Implantology and Oral Radiology, School of Dentistry, Faculty of Health Sciences, Aristotle University of Thessaloniki, Thessaloniki, GRC; 2 Oral Medicine/Pathology, Aristotle University of Thessaloniki, Thessaloniki, GRC

**Keywords:** oral mucosal lesions, differential diagnosis, ossifying fibroma, fibroma, peripheral ossifying fibroma (pof)

## Abstract

A peripheral ossifying fibroma (POF) is a benign, localized lesion that originates from the periosteum or periodontal ligament after traumatic or calculus irritation. The lesions typically manifest in females throughout their second and third decades of life. The diagnosis of a POF is challenging from both clinical and histological standpoints, as it exhibits overlapping features with numerous other clinical entities. This case describes an unusual occurrence of POFs in the anterior maxilla of a 66-year-old female patient who is edentulous at this jaw, but the last two teeth of the lower jaw affect it. The radiographic evaluation revealed no discernible alterations within the bone structure. The diagnosis of POFs was determined through histological investigation. The microscopic examination revealed scattered immature osteoid dystrophic calcified depositions in deep positions, whereas the overlying stratified squamous epithelium manifested frictional keratosis (hyperplasia). The stromal fibroblasts of the collagenous stroma displayed ovoid, normochromatic nuclei, without atypia. Interestingly, the particular importance of this POF case indicates the possibility of an atypical formation in terms of age and location suggesting the role of local chronic irritation as the most critical parameter. Regardless of the initial causative factor, which may be the remnants of the periodontal ligament, the periosteum, or the gingival fibroblasts, ultimately mechanical trauma constitutes the crucial prerequisite so that reactive hyperplasias may be induced.

## Introduction

A peripheral ossifying fibroma (POF) is a non-neoplastic gingival growth that is considered to be of reactive origin. The POF accounts for 2% of all oral lesions [1]. The initial documentation is attributed to Menzel in 1872 [2]. The term "ossifying fibroma" was initially given by Montgomery in 1927 [1]. Additional terminology utilized to describe the POF include a peripheral cementifying fibroma, a peripheral fibroma with cementogenesis, a peripheral fibroma with osteogenesis, a peripheral fibroma with calcification, calcified or ossified fibrous epulis, and calcified fibroblastic granuloma [3,4]. The diagnosis of the POF is challenging due to its intricate clinical and histological features, which exhibit similarities with various different disorders, including fibroma, inflammatory fibrous hyperplasia, pyogenic granuloma, peripheral giant cell granuloma, and non-bone metastases of some cancers [5,6]. The aim of this study is to present a rare case of a maxillary POF, where the last two teeth of the mandible irritated the edentulous maxilla, triggering the formation of the POF.

## Case presentation

**Figure 1:**
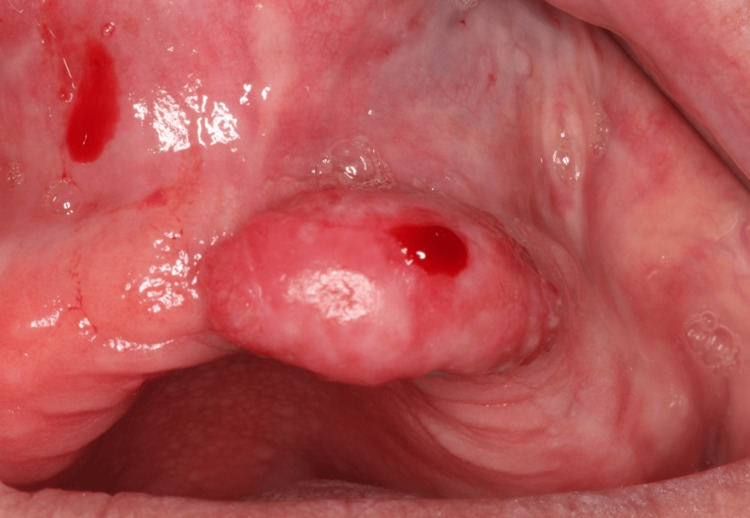
Initial clinical appearance of the tumor.

A 66-year-old female patient was referred to the Department of Dentoalveolar Surgery, Surgical Implantology and Radiology, School of Dentistry, Aristotle University of Thessaloniki, Greece, complaining about the presence of a tumor in the upper jaw, opposite of the last two remaining teeth in the mandible. Before the examination, the patient provided written informed consent. This form was approved by the School of Dentistry, Aristotle University of Thessaloniki and was in accordance with the Helsinki Declaration for research and patient’s ethics. Subsequently, the patient was examined thoroughly. The clinical examination revealed a pedunculated tumor, with hard, slightly elastic in palpation and a slightly whitish discoloration of the surface (Figure 1), exactly opposite of the last two remaining teeth in the mandible (which most probably caused chronic trauma to the edentulous maxillary area, during the occlusion).

**Figure 2:**
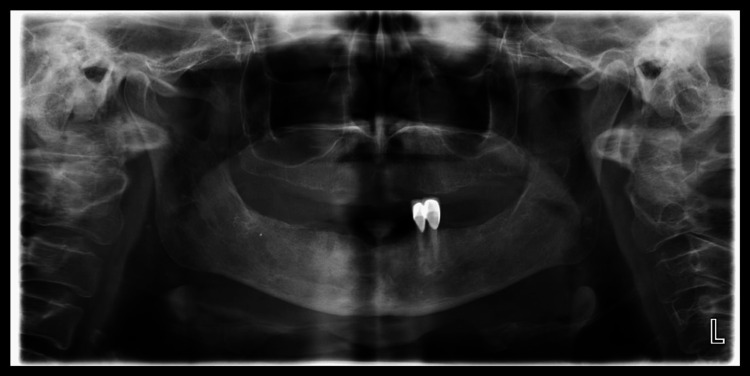
OPG examination of the patient. OPG: Orthopantomography

The orthopantomography (OPG) examination did not reveal any involvement of the maxillary bone (Figure 2).

**Figure 3:**
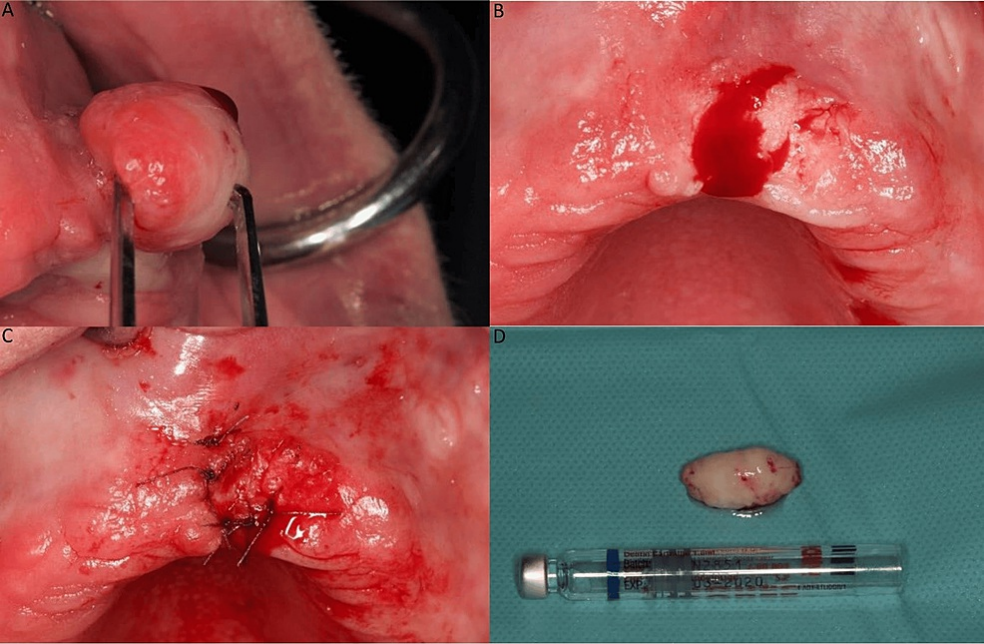
A: Clinical appearance of the tumor. B: Postremoval surgical bed. C: Sutures in place. D: Size of the excised tumor.

The surgical excisional biopsy took place in the Department of Dentoalveolar Surgery, Surgical Implantology and Radiology completed by the use of sutures. The stepwise surgical procedure is displayed in Figure 3.

**Figure 4:**
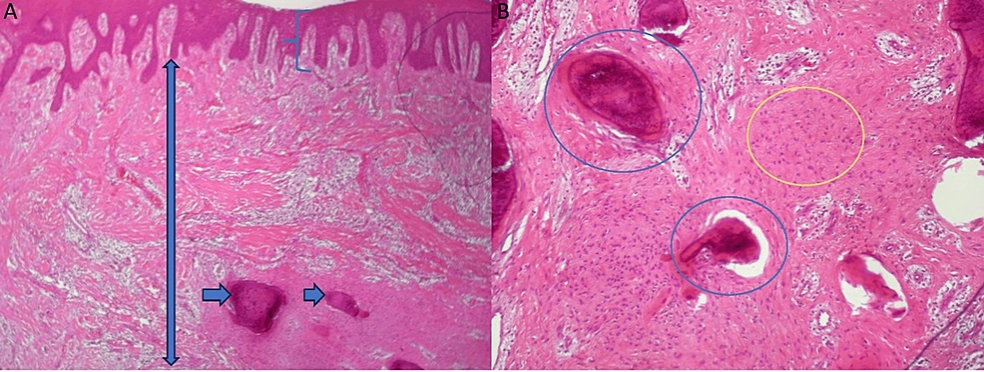
A: The blue bracket shows the epithelium, the double-edged blue arrow shows the connective tissue, and the blue arrows show the osteoid (hematoxylin-eosin stain X40). B: The blue circles show the osteoid and the yellow circle shows the fibroblasts (hematoxylin-eosin stain X100).

The tissue specimen was preserved in formaldehyde and submitted to histopathological examination in the Department of Oral Medicine/Pathology of the same Institution. The microscopic examination revealed scattered immature osteoid dystrophic calcified depositions in deep positions, whereas the overlying stratified squamous epithelium manifested frictional keratosis (hyperplasia). The stromal fibroblasts of the collagenous stroma displayed ovoid, normochromatic nuclei, without atypia (Figure 4).

The clinical and histological findings settled the diagnosis of POF. 

## Discussion

POF development is associated with the periodontal ligament or periosteum, and its location is limited to the soft tissues positioned above the alveolar crest [5]. In our case, the manifestation of the lesion may be attributed to periodontal ligament remnants or periosteum since the patient is edentulous. The most interesting characteristic of this case was the clear association with the local irritation caused by the last two remaining teeth in the mandible. Regardless of the initial causative factor, which may be the remnants of the periodontal ligament, the periosteum, or the gingival fibroblasts, ultimately mechanical trauma constitutes the crucial prerequisite so that reactive hyperplasias may be induced. The prevalence of POFs is higher in females compared to males, and it is frequently observed in the anterior maxilla. Therefore, the epidemiological features of our case are in accordance with the literature. Its occurrence is observed across various age groups, with a higher prevalence observed during the second decade [3]. Out of 657 confirmed cases of POFs, only 134 cases (20%) were observed in the pediatric population aged between 0 and 19 years [1]. Furthermore, it was observed that 8% of these cases occurred within the first decade of life. The POF is commonly observed as an individual, gradually developing nodular formation, either pedunculated or sessile. The external layer of POF typically has a smooth texture, displaying normal color or erythema or red discoloration. Ulceration or frictional keratosis may be present in case of trauma. The majority of ulcerated lesions are observed in patients during their second decade of life [7]. The diagnosis relies on histological analysis, which involves the identification of cellular connective tissue (in particular, unencapsulated accumulation of cellular fibroblastic connective tissue derived from mesenchyme) and the localized presence of bone or other calcifications. This tissue is overlaid with stratified squamous epithelium, which is shown to be ulcerated in around 23-66% of cases [7]. Surgical intervention is considered the preferred therapeutic approach, despite its associated recurrence rate of 20% [1]. POFs may also manifest as benign bone tumors, affecting the jaws. It consists of fibrous connective tissue, with varying degrees of mineralization. The lesion is typically surrounded by a capsule, which helps differentiate it from fibrous dysplasia, a condition that may present with similar clinicopathological characteristics. While the lesion is primarily observed in the jaws, it can also be present in other areas such as the frontal, ethmoid, sphenoid, and temporal bones or orbit, as well as in the anterior cranial fossa [8]. While the average diameter of these lesions is often less than 2 cm, their size can vary significantly. The range of sizes reported is from 0.2 cm to 3.0 cm and from 2 mm to 8 cm [1,9]. Furthermore, there have been cases when lesions have reached a diameter as high as 9 cm [10]. The female-to-male ratios range from 1.22:1 to 4.3:1 [1]. The ethnicity plays also a role, since lighter skinned patients exhibit a higher prevalence of POF, whereas Latin patients exhibit a lower prevalence of POFs [1]. The proliferation of endothelial cells and inflammation can be extensive in regions of ulceration, leading to potential confusion in clinical diagnosis, since the lesion may exhibit similarities to a pyogenic granuloma. The process of mineralization exhibits variability, encompassing cementum-like substance, bone (both woven and lamellar), and dystrophic calcification and it is not related to the size of the lesion [1,7]. The POF lesion is often of limited size and does not necessitate imaging modalities beyond the standard OPG examination and/or intraoral x-ray. The treatment approach involves the conservative surgical removal and scaling of the teeth in close proximity. The latter doesn’t apply in edentulous patients, as in our case. The postsurgical recurrence rates range from 8.9% to 16% [1]. Consequently, it is necessary to engage in regular follow-up.

## Conclusions

In summary, it is a challenge in clinical practice to distinguish between various reactive gingival lesions, especially during the early phases. Irrespective of the specific surgical approach utilized, it is imperative to address the underlying causes and conduct histological analysis of the tissue to validate the diagnosis. Regardless of the initial causative factor, which may be the remnants of the periodontal ligament or the periosteum or the gingival fibroblasts, ultimately mechanical trauma constitutes the crucial prerequisite so that reactive hyperplasias may be induced.
